# Predicting of Daily PM_2.5_ Concentration Employing Wavelet Artificial Neural Networks Based on Meteorological Elements in Shanghai, China

**DOI:** 10.3390/toxics11010051

**Published:** 2023-01-03

**Authors:** Qingchun Guo, Zhenfang He, Zhaosheng Wang

**Affiliations:** 1 School of Geography and Environment, Liaocheng University, Liaocheng 252000, China; 2 Institute of Huanghe Studies, Liaocheng University, Liaocheng 252000, China; 3 State Key Laboratory of Loess and Quaternary Geology, Institute of Earth Environment, Chinese Academy of Sciences, Xi’an 710061, China; 4 State Key Laboratory of Urban and Regional Ecology, Research Center for Eco-Environmental Sciences, Chinese Academy of Sciences, Beijing 100085, China; 5 Ecosystem Science Data Center, Key Laboratory of Ecosystem Network Observation and Modeling, Institute of Geographic Sciences and Natural Resources Research, Chinese Academy of Sciences, Beijing 100101, China

**Keywords:** PM_2.5_, wavelet, artificial neural network, predicting, DNN, CNN, LSTM, COVID-19, epidemic

## Abstract

Anthropogenic sources of fine particulate matter (PM_2.5_) threaten ecosystem security, human health and sustainable development. The accuracy prediction of daily PM_2.5_ concentration can give important information for people to reduce their exposure. Artificial neural networks (ANNs) and wavelet-ANNs (WANNs) are used to predict daily PM_2.5_ concentration in Shanghai. The PM_2.5_ concentration in Shanghai from 2014 to 2020 decreased by 39.3%. The serious COVID-19 epidemic had an unprecedented effect on PM_2.5_ concentration in Shanghai. The PM_2.5_ concentration during the lockdown in 2020 of Shanghai is significantly reduced compared to the period before the lockdown. First, the correlation analysis is utilized to identify the associations between PM_2.5_ and meteorological elements in Shanghai. Second, by estimating twelve training algorithms and twenty-one network structures for these models, the results show that the optimal input elements for daily PM_2.5_ concentration predicting models were the PM_2.5_ from the 3 previous days and fourteen meteorological elements. Finally, the activation function (tansig-purelin) for ANNs and WANNs in Shanghai is better than others in the training, validation and forecasting stages. Considering the correlation coefficients (R) between the PM_2.5_ in the next day and the input influence factors, the PM_2.5_ showed the closest relation with the PM_2.5_ 1 day lag and closer relationships with minimum atmospheric temperature, maximum atmospheric pressure, maximum atmospheric temperature, and PM_2.5_ 2 days lag. When Bayesian regularization (trainbr) was used to train, the ANN and WANN models precisely simulated the daily PM_2.5_ concentration in Shanghai during the training, calibration and predicting stages. It is emphasized that the WANN1 model obtained optimal predicting results in terms of R (0.9316). These results prove that WANNs are adept in daily PM_2.5_ concentration prediction because they can identify relationships between the input and output factors. Therefore, our research can offer a theoretical basis for air pollution control.

## 1. Introduction

Air pollution affects global climate change, ecosystem and human health [1,2,3,4,5]. Additionally, air pollution also leads to huge losses in human capital, productive forces and social welfare [6]. Air pollution is responsible for millions of deaths all over the world [7]. Exposure to air pollution resulted in 7 million premature deaths all over the whole world in 2019 [8]. In total, 1.42 million deaths in China were ascribed to outdoor air pollution in 2019 [9]. High concentration of PM_2.5_ are associated with reduced visibility, economic loss and passive impact on public health because it adds to the incidence rate and mortality of some diseases [10]. Global exposure to environmental PM_2.5_ causes about ~6 to ~10 million deaths every year [6]. In total, 2.12 million deaths in China in 2017 were attributed to PM_2.5_ [11]. Therefore, the accurate prediction of future air pollution can offer reference for travel mode.

With the rapid development of China’s economy, a lot of fossil energy is consumed. Meanwhile, a large number of polluting gases and particulate matter are emitted into the air, seriously influencing the air on which human beings depend. Air pollution is mainly caused by abundant pollutant emissions [12]. It is also strongly related to meteorological conditions [13,14]. The removal and dissipation of air pollutants is determined by the atmospheric diffusion conditions and the precipitation [15].

The outbreak of the COVID-19 pandemic has had a negative impact on social and economic development and human health [16,17]. On 30 January 2020, the WHO Emergency Committee designated COVID-19 as a global health emergency; as of 12 August 2022, COVID-19 had caused at least 585,950,085 confirmed cases and 6,425,422 deaths globally (WHO). The COVID-19 epidemic has had an unprecedented influence on global air pollution [18,19,20,21,22,23]. The abrupt COVID-19 pandemic offers a chance to research the impact of urban blockade policies on the change of air pollutants, and to describe the normal modes of air pollution under the disappearance of the epidemic [24]. The COVID-19 epidemic has had an unprecedented influence on the air pollution in the Beijing and Tianjin districts [25]. During the period of the COVID-19, different urban blockade policies significantly improved the air quality of all four mega cities in China [26]. The reduction of social and productive activities during the lockdown plays an extraordinarily significant role in improving air quality [27]. The implementation of travel restrictions greatly reduced air pollution in 44 cities in China. In addition, the concentration of PM_2.5_ decreased by 5.93% [28]. Air quality can be improved by emphasizing the importance of green commuting, green production and consumption and reducing unnecessary personal trips.

Air pollution forecasting techniques include numerical models and statistical models [29]. The numerical models achieve the simulation of the transformation and diffusion of air pollutants and reflect the change law of air pollutants. However, they are based on a large amount of meteorological information, air pollutant discharge source data and atmospheric monitoring data, they need to master the mechanism of pollution change, and the calculation time is long [30]. Daily PM_2.5_ concentration prediction is a nonlinear, multivariable problem with strong coupling between predictors, so PM_2.5_ numerical forecasting will be an extraordinarily complex system engineering problem. Statistical models are widely used in operational prediction, with the strong points of easy calculation, low data requirements and high precision. Nevertheless, most statistical models align with linear regression theory; assuming that there is nonlinear relationship between pollutant concentration and weather conditions, linear regression is difficult to be applied to nonlinear strongly coupled systems [31]. So far, the artificial intelligence (AI) technique has been extensively applied in a variety of research areas [32,33,34,35].

Machine learning (ML) is an important branch discipline of AI which has been extensively utilized in many research areas [36]. The chief aim of ML is to automatically optimize the nature of algorithms through emulating historical data. ML is able to build steady models, learning from historical data, and utilize these models to forecast future data. Machine learning methods, such as artificial neural networks (ANNs) [37], support vector machines (SVMs), and extreme gradient boosting (XGBoost), have shown fine performance in dealing with nonlinear problems. Deep learning (DL) has also been extensively applied in various fields [38,39]. For example, ordinary recurrent neural networks (RNNs) and convolutional neural networks (CNNs) are usually employed to predict air pollution [40].

Previous researchers have put forward different ML algorithms used for data modeling. Some researchers have proved that ANN has good learning efficiency and is extensively utilized in forecasting groundwater level [41], the COVID-19 epidemic [42], air pollution [43,44], and so on. There is good similarity in the predictive and metrical PM_2.5_ for training in the ANN [45].

Although the deep neural network is powerful, it still has many shortcomings. First, there are too many parameters in DNN, and the learning performance depends heavily on careful parameter adjustment. Secondly, the training of DNN requires a large amount of training data, so it is laborious to apply DNN to tasks with only small-scale training data [46]. In addition, the challenges faced by DL are more common, such as the deficiency of theoretical basis, the insufficiency of interpretability of models, and the need for big amounts of computing resources [47].

There are various types of mother wavelet functions. According to the diurnal variation of air pollutant concentration, each wavelet has its pros and cons in the air pollutant concentration decomposition properties [48]. Using wavelet transform to transform highly variable air pollutant concentrations into several low variability subsequences has distinct merits. For most models, wavelet transform is an effective technique to increase the forecasting accuracy [49]. The basic prediction model uses wavelet transform to decompose the air pollutant concentrations, and then uses artificial neural networks to predict it.

This paper proposes a hybrid model of wavelet transform and ANN (WANN) solution to the problem of predicting the daily PM_2.5_ concentration. To avoid overfitting, the improved algorithms are utilized for modeling, such as trainbr and trainlm. The hybrid model provides a novel alternative for forecasting daily PM_2.5_ concentration.

## 2. Materials and Methods

### 2.1. Study Location and Data Sources

**Figure 1:**
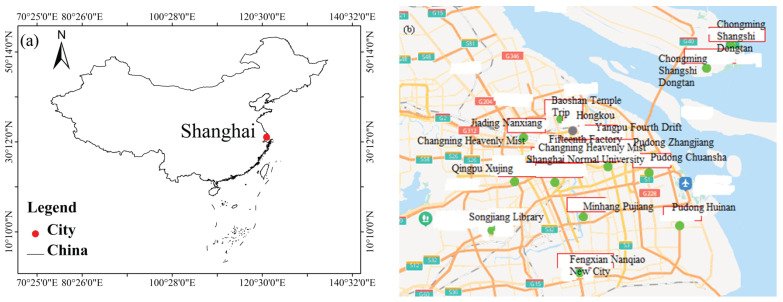
The geographical position of Shanghai city in China. (**a**) The location of Shanghai city in China; (**b**) the location of the monitoring sites in Shanghai city.

Shanghai is the largest city in China (Figure 1a). Shanghai is located in East China with the area of 6340 km^2^, and it is at the estuary of the Yangtze River. The average altitude of Shanghai is 2.19 m, and the permanent resident population and the GDP in 2021 were about 24.8943 million and CNY 4321.485 billion.

In this paper, the air pollution data sets and meteorological data sets of Shanghai from 1 January 2014 to 31 December 2020 are utilized. The daily PM_2.5_ concentration data are from the mean values of twenty monitoring sites (stations) in Shanghai and can be obtained on the website of China Environmental Monitoring Station (http://www.cnemc.cn/) (accessed on 21 January 2022) and platform (http://www.aqistudy.cn/) (accessed on 22 January 2022) (Figure 1b). Table 1 displays the list of the monitoring stations used in this study. The data of meteorological elements (including temperature, precipitation, humidity, wind, atmospheric pressure, etc.) are from the average value of the observation station of the China Meteorological Administration. These data are divided into three stages, namely, the training stage (80%), the verification stage (10%) and the prediction stage (10%). The training stage is from 1 January 2014 to 30 June 2019, the verification stage is from 1 July 2019 to 31 March 2020, and the prediction stage is from 1 April 2020 to 31 December 2020.

### 2.2. Wavelet Transformation (WT)

**Figure 2:**
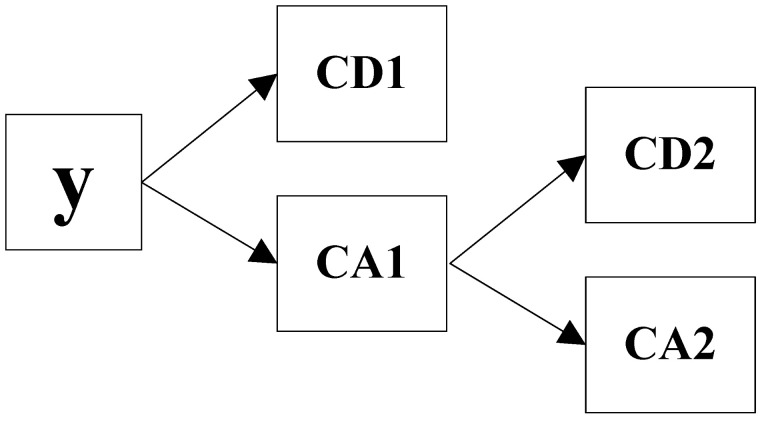
Schematic diagram of 2-level wavelet decomposition.

Wavelet transformation (WT) is one of the waveform analytical methods for time-varying signals. In wavelet transform, the wavelet coefficients can be obtained by convolution integration of the mother wavelet function and the original time domain signal. Discrete wavelet transform (DWT) has the advantage of less computational expense than continuous wavelet transform (CWT). The Daubechies (db) wavelet is the most commonly utilized mother wavelet function. The Mallat pyramidal algorithm is used to compute DWT. Therefore, the DWT is used to decompose the daily PM_2.5_ concentration data and meteorological elements data [50]. The DWT of a time series f(q) is defined as Equation (1):

(1) $$f(c,d)=\frac{1}{\sqrt{c}}\int_{-\infty }^{\infty }f(h)\psi (\frac{h-d}{c})dh$$

where *ψ*(*h*) expresses the fundamental wavelet of effective length *h*; *c* expresses the scale or dilation factor; and *d* expresses the translation time. For a discrete signal y, the DWT is defined by multi-resolution decomposition, which can be computed by the Mallat decomposition algorithm and Mallat pyramidal reconstruction algorithm [41]. For m-level decomposition and reconstruction, the original signal y can be expressed as

(2) $${y=\mathrm{CA}}_{m}+\sum_{i=1}^{m}CD_{i}$$

where CA*m* is the approximation series representing the low-frequency component, which contains trend information, and *CDi* is the detail series on the *i* level representing the high-frequency component, which contains periodic information. Basically, this is a process in which the low-frequency sequence is decomposed into low-frequency subsequences and relatively high-frequency subsequences with the increase in m (Figure 2). The results of the 2-level wavelet decomposition of the original time series of PM_2.5_ concentration by applying bior1.1 wavelets was implemented in the wavelet toolbox of MATLAB.

The main purpose of utilizing the discrete wavelet transform is to reduce the complexity of the input signal and the amount of relevant information between the decomposition combinations (detailed CD2, CD1 and approximate CA2). Discrete wavelet transform could be used to approximate components to obtain low dimensional components and gain components for multidimensional analysis.

### 2.3. Artificial Neural Network (ANN)

**Figure 3:**
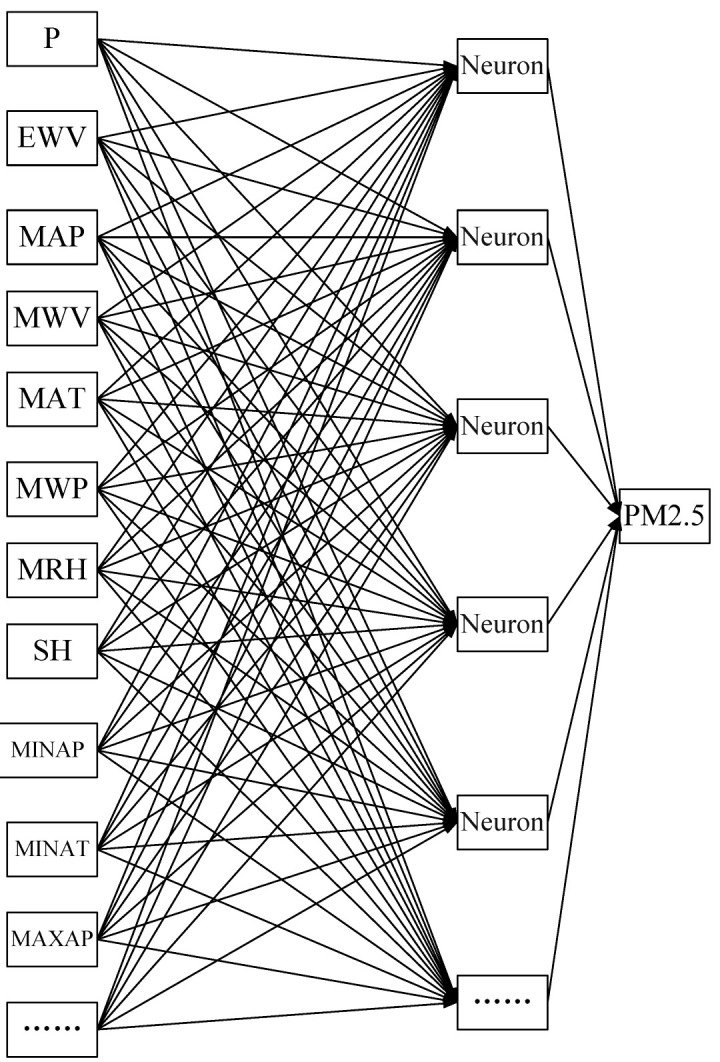
Artificial neural network architecture for mean daily PM_2.5_ concentration forecasting.

An artificial neural network (ANN) is a part of AI. It simulates the prediction and recognition functions of the biological brain and is used to solve complex problems in various application fields. The typical network architecture of an ANN consists of three layers (i.e., input layer, hidden (implication) layer and output layer), each one composed of several artificial neurons and an activation function. Each artificial neuron is contacted via weights and gains information from the correlative neurons for processing. Owing to its strong nonlinear processing features, ANN could output nonlinear relationships of many complicated scientific problems. The proposed ANN model for predicting the daily PM_2.5_ concentration is displayed in Figure 3. The seventeen input neurons of the input layer are designed as the key operating parameters, which include precipitation (P), extreme wind velocity (EWV), mean atmospheric pressure (MAP), mean wind velocity (MWV), mean atmospheric temperature (MAT), mean water vapor pressure (MWP), mean relative humidity (MRH), sunshine hours (SH), minimum atmospheric pressure (MINAP), minimum atmospheric temperature (MINAT), maximum atmospheric pressure (MAXAP), maximum atmospheric temperature (MAXAT), maximum wind velocity (MAXWV), minimum relative humidity (MINRH), PM_2.5_ (t), PM_2.5_ (t − 1), and PM_2.5_ (t − 2).

Back propagation (BP) is the most commonly used and effective method to train the artificial neural network (ANN) algorithm. In the process of model development, there are two phases of forward propagation and error back propagation. The hidden (implication, middle) layer neurons calculate the weighted summation of the acquired input layer information s using Equations (3) and (4), and transmit these to the coming layer through the activation function (transfer function), then contrast the error criterions between the input value and the metrical value, then transfer the error back to the input layer, and decrease the error to the goal standard by altering the relation weight and thresholds (deviations or biases) [51].

(3) $$k=\sum_{i=1}^{m}w_{ij}O_{i}+p$$

(4) Q=f(k)

where *k* is the weighted total, *w_ij_* is the relation weight, *j* is the number of neurons in the output layer, *O_i_* is the input data, and *p* is the biases (deviation or thresholds) value, utilized to balance the effect of the activation function. *Q* is the output data, and *f* is the activation function. After the forward propagation transversion of the signals, the global error is counted. If the global error is lower than the setting error (10^−5^), the backward propagation of the global error is completed to change the weights and thresholds. The back propagation of the global error function is counted as in Equation (5):

(5) $$E=\frac{1}{l}{\sum_{j=1}^{l}\left(T_{j}-Q_{j}\right)}^{2}$$

where *E* is the error of the current output, *T_j_* is the target output, *Q_j_* is the predicted output, and l is the total output number (2004). After adjusting and training the network model, the messages of the input parameters could be stored for modelling, such as weights and thresholds (biases).

Four kinds of activation functions are usually utilized in BPANN are *sigmoid (logsig), tanh (tansig), purelin and ReLU (poslin)* functions, which are logarithmic sigmoid, hyperbolic tangent sigmoid, linear, and positive linear transfer functions, respectively. The four functions of the network are defined as follows:

(6) $$sigmoid(r)=\frac{1}{1+e^{-r}}$$

(7) $$tansig(r)=\frac{e^{r}-e^{-r}}{e^{r}+e^{-r}}$$

(8) purelin(r)=r

(9) $$ReLU(r)=\left\{{}_{0,if(r\leq 0)}^{{}^{r,if(r\geq 0)}}\right.$$

where *r* is the corresponding input.

Artificial neural networks could fulfil well in the training information, but not well in the forecasting information, which explains that they perform poorly as different information or error increases. When the artificial neural network (ANN) cannot generalize this problem, it is called “overfitting”. This problem could be solved utilizing the Bayesian regularization algorithm (BR, or trainbr), Levenberg–Marquardt algorithm (LM, or trainlm) or other training algorithms [52]. Trainbr is a function which updates weights and threshold (bias or deviation) values on the basis of LM optimization. It minimizes the union of square error and weight, and then ascertains the correct union to generate a network with good generalization. In addition, the LM algorithm (trainlm) is a variant of Newton’s way, which is devised to minimize the sum of squares of other nonlinear functions. While the property function has the modality of the summation of squares, the Hessian matrix could be calculated approximately as the outcome of the Jacobian matrix, which is much less complicated than calculating the Hessian matrix.

The raw data were normalized, for quick convergence, and rendered dimensionless. The results after treatment are as follows:

(10) $$S=\frac{s-s_{\min}}{s_{\max}-s_{\min}}$$

where *S* is the normalized data for the original variable, *s*_min_ is the minimum of the raw data, *s*_max_ is the maximum of the raw data, and *s* denotes the original data.

### 2.4. Wavelet Artificial Neural Network

**Figure 4:**
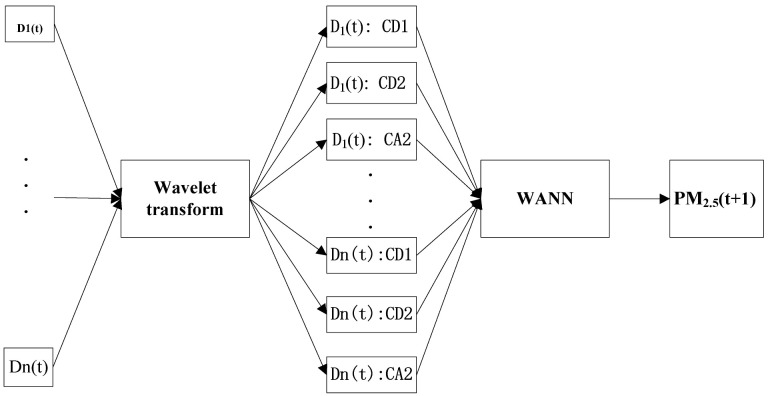
Model structure of wavelet artificial neural network for mean daily PM_2.5_ concentration forecasting.

The WANN model is utilized to decompose the raw data Dn (t) into three suites: CD2, CD1 and CA2. After that, these data are employed by the ANN as the input factors. In Figure 4, Dn (t) is the input factors of day t, PM_2.5_ (t + 1) is the PM_2.5_ predicted t + 1 day in the future.

### 2.5. Performance Criteria

Three kinds of statistical indicators were adopted to appraise the nature of ANN and WANN models. These are mean absolute error (*MAE*), root mean square error (*RMSE*), and correlation coefficient (*R*), which are as follows:

(11) $$MAE=\frac{1}{U}\sum \left|A_{k}-C_{k}\right|$$

(12) $$RMSE=\sqrt{\frac{\sum {\left(A_{k}-C_{k}\right)}^{2}}{U}}$$

(13) $$R=\frac{\sum \left(A_{k}-\overset{-}{A})(C_{k}-\overset{-}{C}\right)}{\sqrt{\sum {\left(A_{k}-\overset{-}{A}\right)}^{2}{\left(C_{k}-\overset{-}{C}\right)}^{2}}}$$

*A_k_* expresses the *k*th observed PM_2.5_ concentration, *C_k_* expresses the *k*th predicted PM_2.5_ concentration, $\overset{-}{A}$ is the mean of the observed PM_2.5_ concentration, $\overset{-}{C}$ is the mean of the predicted PM_2.5_ concentration, and *U* is the number of observed PM_2.5_ concentration.

## 3. Result and discussion

### 3.1. Long Term Change of PM_2.5_ Concentration in Shanghai

**Figure 5:**
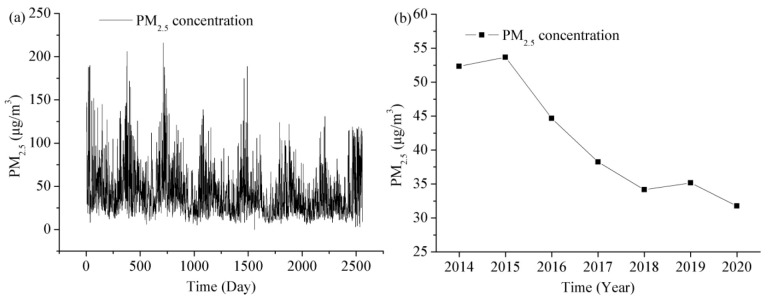
The mean daily and annual variations of the original PM_2.5_ concentration data during the study period 2014–2020. (**a**) Daily PM_2.5_ concentration data, (**b**) annual PM_2.5_ concentration in the study area.

As shown in Figure 5, the PM_2.5_ concentration in Shanghai shows a tendency of descending year after year. The annual mean PM_2.5_ concentrations from 2014 to 2020 are 52.33 μg/m^3^, 53.67 μg/m^3^, 44.67 μg/m^3^, 38.25 μg/m^3^, 34.17 μg/m^3^, 35.17 μg/m^3^, and 31.75 μg/m^3^, in the range of 8–190 μg/m^3^, 6–216 μg/m^3^, 5–163 μg/m^3^, 7–175 μg/m^3^, 6–189 μg/m^3^, 6–122 μg/m^3^, and 3–131 μg/m^3^, respectively. The PM_2.5_ concentration in Shanghai decreased by 39.3% from 2014 to 2020. This change improved the PM_2.5_ level from about 10 times the World Health Organization (WHO) guidelines to about 6 times. The average value of PM_2.5_ concentration in Shanghai for the 7 years is 41.43 μg/m^3^. Although the air quality in Shanghai has improved a lot, it exceeded the new Global Air Quality Guidelines (AQGs) of the WHO standard (5 μg/m^3^ above the annual PM_2.5_ limit). The average value change of PM_2.5_ in 7 years has a U-shaped characteristic, with the maximum in January and the minimum in August. The seasonal average value of PM_2.5_ in 7 years has obvious change characteristics, with the maximum in winter, followed by spring, autumn and the minimum in summer.

The COVID-19 epidemic has had a significant impact on the PM_2.5_ concentration in Shanghai. These data in 2019–2020 are divided into three parts: period I (1 January to 26 January, 2019–2020); period II (27 January to 30 April, 2019–2020); and period III (1 May to 31 July, 2019–2020). Period II is the lockdown period. The values of PM_2.5_ during period I, period II, and period III in 2020 are, respectively, 52.62, 32.41, and 31.99 (μg/m^3^). However, those are, respectively, 53.12, 43.11, and 29.58 (μg/m^3^) in the same period of 2019. Compared with those values in 2019, these values of PM_2.5_ in 2020 decreased by 0.5, 10.7, and −2.41 (μg/m^3^), which are respectively 0.9, 24.8, and −8.2% lower in 2020 than those in 2019. The air quality during lockdown in 2020 is apparently improved compared with that in the same period of 2019.

### 3.2. Relevance between Daily PM_2.5_ Concentration and Meteorological Factors in Shanghai

**Figure 6:**
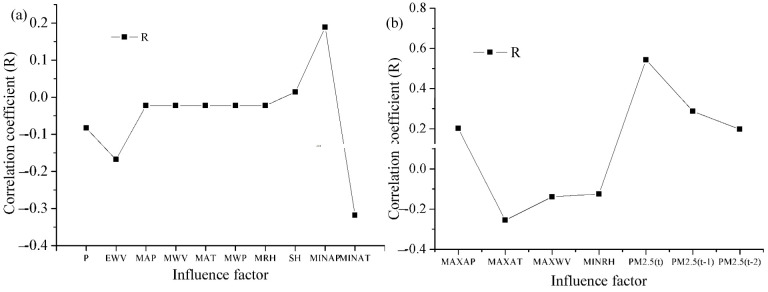
Relevance between daily PM_2.5_ (t + 1) and input factors. (**a**) Correlation coefficient between meteorological elements and PM_2.5_ (t + 1). (**b**) Correlation coefficient between meteorological elements, PM_2.5_ (t) and PM_2.5_ (t + 1).

Correlation analysis could ascertain the linear relationships between PM_2.5_ concentration and meteorological elements. The determination of input variables is one of the most significant parts in the projection of ANN and WANN models. The results of the relationships counted for the input factors are shown in Figure 6, which is significant at the 0.01 level (2-tailed).

The correlation between each factor and PM_2.5_ (t + 1) was appraised by determining its R. The analysis results exhibited that PM_2.5_ (t) was strongly related to PM_2.5_ (t + 1) in Shanghai. In addition, the performance results of MINAT, MAXAP, MAXAT, PM_2.5_ (t), PM_2.5_ (t − 1) were better than other factors in Shanghai. That is, these meteorological factors have the highest correlation to PM_2.5_ (t + 1). We ascertained five important factors. Therefore, various combinations of factors were used as inputs for simulating daily PM_2.5_ (t + 1) in Table 2. For example, the network structure 17:5:1 in Table 1 indicates that there are 17 neurons in the input layer, 5 neurons in the hidden layer, and 1 neuron in the output layer. Other network structures are similar. The ascertaining factors were chosen based on relationship with PM_2.5_.

### 3.3. Determination of Model Structure and Parameters

**Figure 7:**
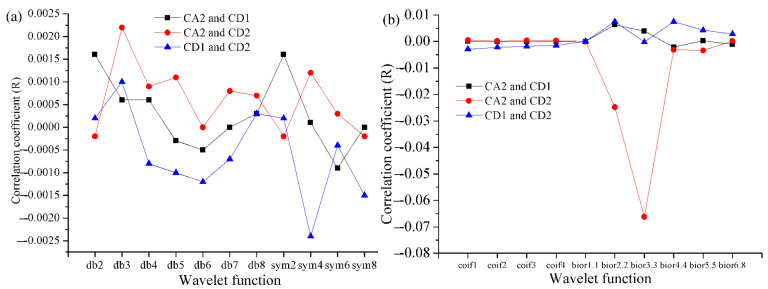
Correlation between CA2, CD1 and CD2 for distinct wavelet functions (mother wavelets) in Shanghai during the testing phase; (**a**) wavelet Daubechies (db) and symlets (sym); (**b**) wavelet coiflets (coif) and biorthogonal wavelets (bior). Bior6.8 is a biorthogonal wavelet with an even symmetric high-pass decomposition filter.

It should be stressed that selecting the most compatible network structure is one of the important assignments of the model designer. The important information obtained from meteorological elements is extracted by discrete wavelet transform (DWT). The various details and dimensions of input factors are gained by two-period decomposition of WT. After two-period decomposition and reconstruction, the input factors are changed into three parts. The approximate component CA2 represents the low frequency information of the raw factor, while the detailed CD2 and CD1 represent the high-frequency information of the raw factor. The change characteristics of time series are the key factors affecting wavelet selection [53]. In order to optimize the decomposition of input factors, the mother wavelet is chosen, and the correlation between CD1, CD2 and CA2 is considered. The minimum R could primely meet the objective of analyzing the change characteristics of various components of input factors. The quantitative estimation showed that the components were independent of one another. In total, 21 types of wavelet functions are ascertained for wavelet transform. The mother wavelets (wavelet functions) evaluated are Daubechies (db), symlets (sym), coiflets (coif), and biorthogonal wavelets (bior) [54]. Coiflets are a family of compactly supported orthogonal wavelets. Figure 7 shows that bior1.1 is the best wavelet function in the current research on account of the smallest R. bior1.1 is a biorthogonal wavelet [54]. The correlation coefficients between CD1, CD2 and CA2 after the input factors are decomposed by bior1.1 are all 0.

**Figure 8:**
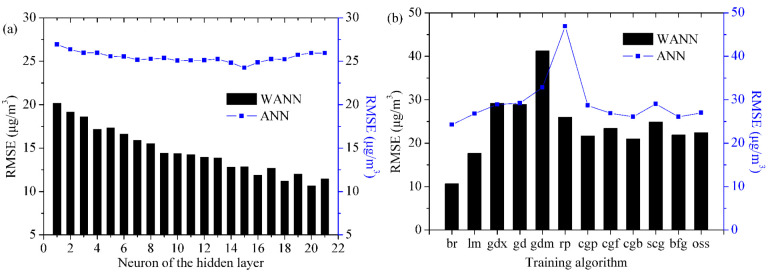
Performance comparison of different ANN and WANN structures and training algorithms in Shanghai during the testing phase. The training algorithms include trainbr (br), trainlm (lm), traingdx (gdx), traingd (gd), traingdm (gdm), trainrp (rp), traincgp (cgp), traincgf (cgf), traincgb (cgb), trainscg (scg), trainbfg (bfg), and trainoss (oss). (**a**) Performance of different nodes in hidden layer for ANN and WANN. (**b**) Performance of different training algorithms for ANN and WANN.

The optimal model parameters are obtained by the trial-and-error method. Figure 8 demonstrates that the network structures (17-15-1 for ANN and 51-20-1 for WANN) are superior to other network topologies through repeated tests. In the models, the number of neurons of the hidden (implication, middle) layer increases from 1 to 21. It can be seen from Figure 6 that the RMSE value decreases slightly with the increase in the number of hidden (implication, middle) layer neurons. Consequently, the optimal structures of the mode for Shanghai are 17-15-1 (ANN) and 51-20-1 (WANN), respectively.

Figure 8 expresses the properties of the training algorithms, indicating that the trainbr algorithm has the best property in forecasting PM_2.5_ (t + 1) in Shanghai. Trainbr automatically fits the optimal values of the objective function parameters.

**Figure 9:**
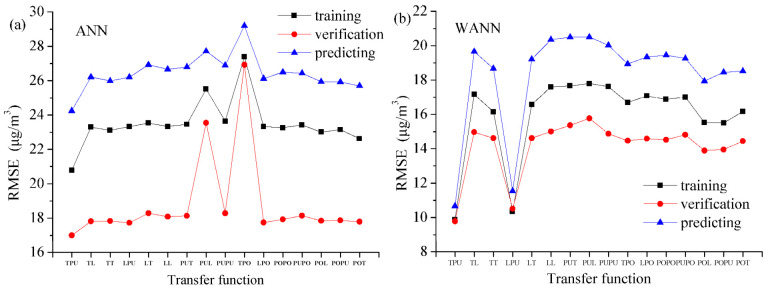
Comparison between various activation functions for ANN and WANN in Shanghai during the training, verification and predicting phase. (**a**) RMSE for ANN, transfer functions include tansig(T), purelin(PU), logsig(L), and poslin(PO); (**b**) RMSE for WANN.

Figure 9 shows that the activation function (tansig-purelin) for ANN in Shanghai is better than others during training, calibration and predicting stages. In the same way, the transfer function (tansig-purelin) for WANN in Shanghai is also better than others in Figure 9.

### 3.4. Comparative Analysis of the Different PM_2.5_ Predicting Models

All results of the ANNs and WANNs during the training, validation and predicting stage are shown in Table 3. We used the ten-fold cross-validation method to verify the models. During the training stage, the root mean square errors (RMSEs) of ANN1 and WANN1 in Shanghai were 20.7841 and 9.8824, respectively; mean absolute errors (MAEs) were 15.0825 and 7.1153, respectively; and correlation coefficients (Rs) were 0.7061 and 0.9416, respectively. In the meantime, RMSE, MAE, and R for ANN2, ANN3, ANN4, WANN2, WANN3, and WANN4 have similar results. During the training stage, the WANNs were superior to the ANNs. During the verification stage, the RMSEs of ANN1 and WANN1 in Shanghai were 17.0006 and 9.7850, respectively; MAEs were 13.1262 and 6.8827, respectively; and Rs were 0.6830 and 0.8969, respectively. During the predicting stage, the RMSEs of ANN1 and WANN1 in Shanghai were 24.2407 and 10.6580, respectively; MAEs were 17.7867 and 7.6918, respectively; and Rs were 0.5618 and 0.9316, respectively. In the above three stages, the WANNs were also superior to the ANNs. The WANN1 model based on all 17 input variables is the best model in predicting PM_2.5_ concentration. The WANN2 model based on five input variables is the second-best model for predicting PM_2.5_ concentration. It is interesting that the performance of WANN2 is similar to WANN1. These two models can meet the PM_2.5_ concentration prediction requirements.

**Figure 10:**
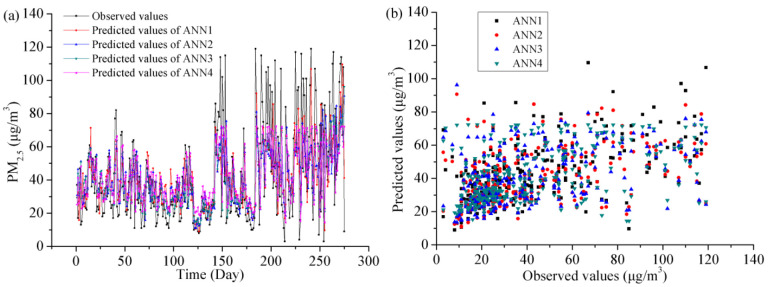
The forecasting outcomes and scatter plots with the ANN models in the testing stage. (**a**) The predicted versus observed PM_2.5_ concentration, (**b**) scatter plots.

Figure 10 displays the forecasting PM_2.5_ outcomes and scatter plots with the ANN models in the testing stage in Shanghai. ANNs were able to replicate the average of the daily PM_2.5_ concentration but were limited in capturing minimal or maximal peaks. However, the predicted and observed values are relatively scattered.

**Figure 11:**
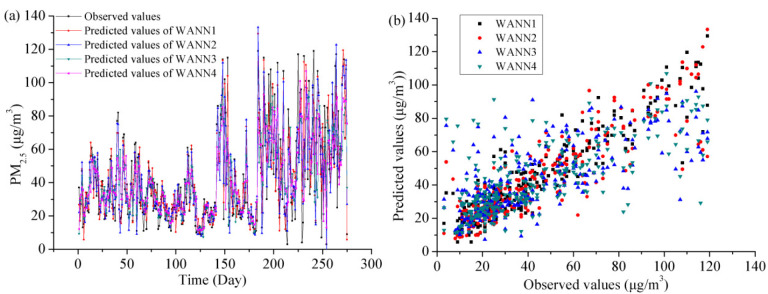
The forecasting outcomes and scatter plots with the WANN models in the testing stage. (**a**) The predicted versus observed PM_2.5_ concentration, (**b**) scatter plots.

Figure 11 indicates the forecasting line and scatter plots with the WANNs in the testing stage. The WANNs predicted daily PM_2.5_ concentration at an acceptable precision level in Shanghai. Additionally, WANNs were apparently superior to ANNs. The WANNs reproduced a good consistency between the observed PM_2.5_ (t + 1) concentration and predicted PM_2.5_ (t + 1) concentration. It is also apparent that the WANN1 model with 14 meteorological elements was better than the WANN4 with 1-day lag PM_2.5_ concentration; in other words, including 14 meteorological elements and the 3 former days’ PM_2.5_ as parameters in the input factors supplies more precise results. The agreement between the observed PM_2.5_ (t + 1) concentration and the predicted PM_2.5_ (t + 1) concentration is also very good in Shanghai using the WANN2 model. The main meteorological elements of the WANN2 model are MINAT, MAXAP, and MAXAT in Shanghai. The possible reason is that the relationship between them and PM_2.5_ is stronger than for other meteorological elements.

### 3.5. Comparison with Other Existing PM_2.5_ Prediction Models

Many ML means have been utilized for PM_2.5_ prediction. Table 4 shows the R^2^, relative errors (REs), RMSE, and MAE of different methods. The value of R^2^ was 0.74 while training the ANN with 90% of basic data [45]. ANN was utilized to predict concentration of PM_2.5_ for the coming 1 day in Delhi, India. Coefficient of correlations for the ANN is 0.65 [55]. The Trainlm using an ANN modeling nicely forecasted the vehicle exhaust emission of PM_2.5_ with the R^2^ of 0.94 in Addis Ababa, Ethiopia [56]. The support vector regression (SVR) and multiple linear regression (MLR) models provide more accurate and reliable predictions than other evaluation models. Among the ML models with the best performance, the execution speed of SVR is about five times that of the MLR model, and the lowest MAE for hourly prediction is 1.294 μg/m^3^ for t_0_ and 3.752 μg/m^3^ for t + 12 [57]. The XGBoost model can accurately predict the daily PM_2.5_ (R^2^ = 0.80, RMSE = 14.75 μg/m^3^) [58]. It is confirmed that the forecasting of the RNN model chiefly depends on the input information. The MAE of the RNN model for PM_2.5_ prediction is 8.4 [59]. The optimized LSTM model has good assessment criteria, with R^2^ = 0.94, RMSE = 13.06 μg/m^3^, and MAE = 8.61 μg/m^3^ [60]. The CNN for PM_2.5_ prediction in Beijing has a R of 0.85, a RMSE of 40.83 μg/m^3^, and a MAE of 25.32 μg/m^3^ [61].

The hybrid models are also widely used in PM_2.5_ prediction. The R-square, RMSE, and MAE of the gated recurrent unit neural network based on the empirical mode decomposition (EMD-GRU) model are, respectively, 0.9852, 11.372 μg/m^3^, and 6.532 μg/m^3^. These values are better than the decision tree regressor (DTR), support vector machine (SVM), random forest (RF), recurrent neural networks (RNNs), gradient boosted decision trees (GBDTs), long short-term memory (LSTM), and gated recurrent unit neural network (GRU). These results prove that the EMD-GRU model has a better simulation result and stronger precision than ordinary ML or DL models [62]. CNN and LSTM are combined and utilized to forecast PM_2.5_ concentration. The R^2^, RMSE, and MAE of CNN-LSTM are, respectively, 0.92157312, 24.22874 μg/m^3^, and 14.63446 μg/m^3^ [63]. The 3D CNN-GRU model was utilized to predict the PM_2.5_ level. Compared with LSTM, ANN, SVR, GRU, and autoregressive integrated moving average (ARIMA), it can obtain promising results; it estimated 78% (R^2^ = 0.78) of PM_2.5_ concentration changes in the coming day [64]. Compared with other related DL or solitary models, the hybrid MCD-ESN-PSO model has better prediction accuracy for PM_2.5_ concentration in four cities of China [65]. Considering CNN and the gradient boosting machine (GBM) method, a mixed model for estimating the PM_2.5_ concentration in Shanghai was established. The constructed CNN-GBM model has good estimation accuracy, with the RMSE of 10.02 [66]. The iDeepAir model can accomplish better simulating and forecasting performance than the Seq2Seq, gradient boosting regression tree (GBRT), dual-stage attention-based recurrent neural network (DA-RNN), LSTM model and other DL models. Specifically, compared to ARIMA, iDeepAir could decrease the MAE from 25.36 μg/m^3^ to 12.28 μg/m^3^ [67]. Compared with the traditional land use regression (LUR) and SVM models, the prediction accuracy of the combined genetic algorithm and support vector machine (GA-SVM) method for PM_2.5_ concentration is significantly improved, with a validation determination coefficient (R^2^) of 0.84, and a lower RMSE and an MAE of 12.1 μg/m^3^ and 10.07 μg/m^3^, respectively [68].

Compared with the outcomes of other PM_2.5_ prediction models, our WANN model is in the upper middle position. Because each model has advantages and disadvantages, and different regions require different models, it is necessary to develop general artificial intelligence. Artificial intelligence could have decision-making processes that are very difficult to explicate with current knowledge. In addition, the application of the R value based on the correlation analysis method for variable selection keep more important information for prediction and shorten the model running time. MINAT(t), MINAP(t), and MAXAT(t) are input parameters for prediction at most levels. The main influencing factors of the low-level detail series are precursors, while the approximation series is affected by meteorological conditions and the accumulated PM_2.5_. The WT method improves the predictive performance of the ANN significantly.

## 4. Conclusions

In this study, we study the ML modeling technology on small data sets. The results prove that WANNs perfected the property of the regression model. Generally, the property of the WANNs was better than that of the ANNs in this work. The training algorithm trainbr avoids overfitting; consequently, a more powerful model could be established. These models have very different numbers of inputs (such as 17 versus 5), so their predicted results are different. When the input variables are the same, they are comparable (such as WANN1 versus ANN1).

The prediction methods of the PM_2.5_ concentration make use of meteorological elements. There is an intimate relation between the meteorological elements and PM_2.5_ concentration. Moreover, the relationship between meteorological elements and PM_2.5_ concentration is nonlinear. The important information obtained from meteorological elements is extracted by discrete wavelet transform (DWT). ANNs and WANNs have flexible mathematical structures and can map highly nonlinear relationships between meteorological elements and PM_2.5_ concentration. The performance results of WANNs are better than those of ANNs in Shanghai. Most WANN models have success in predicting PM_2.5_ concentration.

The severe COVID-19 epidemic has had an unprecedented impact on PM_2.5_ concentration in Shanghai. The air quality in Shanghai during lockdown is apparently better than before lockdown. The air quality during lockdown in 2020 was apparently improved compared with those in the same period of 2019.

We examined the practicability of utilizing artificial intelligence with meteorological elements as input factors to forecast the coming day’s PM_2.5_ concentration. The performance results of the ANNs and WANNs are evaluated using three criteria. A simple WANN model with 17 elements as input variables is used as a reference case. The accurate prediction ability of the WANN model is also proved.

China has formulated the grand goal of carbon neutrality and pollution reduction. In this paper, we only use ANN and WANN for daily PM_2.5_ prediction and consider the meteorological elements and PM_2.5_ concentration of the last 3 days as predictors. In the future, in order to further improve the effectiveness of future forecasting, we will use deep learning and hybrid models to predict PM_2.5_ concentration in other cities, such as LSTM, CNN, gated recurrent units networks (GRUs), deep belief network (DBN), graph convolutional network (GCN), wavelet-LSTM (W-LSTM), wavelet-GRU (W-GRU), the integration of CNN-LSTM, and Community Multiscale Air Quality (CMAQ-CNN), and consider air pollutant emissions.

## Figures and Tables

**Table 1:** The list of the monitoring stations used in this study in Shanghai city.

Monitoring Sites	Monitoring Sites
Jinshan New City	Minhang Pujiang
Chongming Shangshi Dongtan	Qingpu Xujing
Chongming Shangshi Dongtan	Shanghai Normal University
Yangpu Fourth Drift	Pudong Zhangjiang
Fifteenth Factory	Baoshan Temple Trip
Jing’an Monitoring Station	Fengxian Nanqiao New City
Pudong Huinan	Jiading Nanxiang
Putuo	Songjiang Library
Pudong Chuansha	Changning Heavenly Mist
Pudong New Area Monitoring Station	Hongkou

**Table 2:** Sets of input factors that were tested with the ANN and WANN models for the predicting of next-day PM_2.5_ concentrations in Shanghai.

Model ID	Input Variables	Structure
ANN1 ANN2	P (t), EWV (t), MAP (t), MWV (t), MAT (t), MWP (t), MRH (t), SH (t), MINAP (t), MINAT (t), MAXAP (t), MAXAT (t), MAXWV (t), MINRH (t), PM_2.5_ (t), PM_2.5_ (t − 1), PM_2.5_ (t − 2) MINAT(t), MINAP(t), MAXAT(t), PM_2.5_ (t), PM_2.5_ (t − 1)	17:15:1 5:19:1
ANN3	MINAT(t), PM_2.5_ (t)	2:19:1
ANN4	PM_2.5_ (t)	1:21:1
WANN1	P (t), EWV (t), MAP (t), MWV (t), MAT (t), MWP (t), MRH (t), SH (t), MINAP (t), MINAT (t), MAXAP (t), MAXAT (t), MAXWV (t), MINRH (t), PM_2.5_ (t), PM_2.5_ (t − 1), PM_2.5_ (t − 2)	51:20:1
WANN2	MINAT(t), MINAP(t), MAXAT(t), PM_2.5_ (t), PM_2.5_ (t − 1)	15:20:1
WANN3	MINAT(t), PM_2.5_ (t)	6:17:1
WANN4	PM_2.5_ (t)	3:19:1

**Table 3:** Comparison of the performance statistics using different models.

Model	R			RMSE (μg/m^3^)			MAE (μg/m^3^)		
	Training	Verification	Predicting	Training	Verification	Predicting	Training	Verification	Predicting
ANN1	0.7061	0.6830	0.5618	20.7841	17.0006	24.2407	15.0825	13.1262	17.7867
ANN2	0.6271	0.6258	0.4731	22.8559	18.1992	25.9092	16.3347	14.1125	18.9660
ANN3	0.5947	0.5831	0.4454	23.5883	18.7088	26.4504	16.9128	14.5707	19.2679
ANN4	0.5759	0.5450	0.4244	23.9847	18.9768	26.7117	17.2196	14.7856	19.7467
WANN1	0.9416	0.8969	0.9316	9.8824	9.7850	10.6580	7.1153	6.8827	7.6918
WANN2	0.9075	0.8424	0.8830	12.3243	11.9231	13.7228	8.4519	8.1825	9.1533
WANN3	0.7952	0.6860	0.7213	17.7836	16.1466	20.2332	12.5331	11.1733	14.6255
WANN4	0.7380	0.6404	0.7043	19.7903	17.0581	20.7106	13.5311	11.5643	14.7616

**Table 4:** The difference between existing PM_2.5_ prediction models and our model.

Model	Area	R^2^	RE	RMSE	MAE	Reference
ANN	Ahvaz, Iran	0.74	0.91507	46.44		[45]
ANN	Delhi, India	0.86	0.451			[55]
ANN	Addis Ababa, Ethiopia	0.943	0.12034	15.66	10.27	[56]
SVR	Nottingham, United Kingdom	0.88782	0.12224	2.45315	1.29443	[57]
XGBoost	China	0.8	0.36385	26.34	15.58	[58]
RNN	Seoul metropolitan, Korea		0.31		8.4	[59]
LSTM	Tianjin, China	0.94	0.4305	13.06	8.61	[60]
CNN	Beijing, China	0.7225	0.58843	40.83	25.32	[61]
EMD-GRU	Beijing, China	0.9706	0.14809	11.372	6.532	[62]
CNN-LSTM	Beijing, China	0.921573	0.1518	24.2287	14.63446	[63]
3D CNN-GRU	Tehran, Iran	0.78	0.27781	6.44	8.89	[64]
MCD-ESN-PSO	four cities, China	0.9801	0.0167	1.18	0.88	[65]
CNN-GBM	Shanghai, China	0.85	0.07982	10.02		[66]
iDeepAir	Shanghai, China		0.2227	15.587	12.373	[67]
GA-SVM	Shaanxi, China		0.18773	12.1	10.07	[68]
WANN	Shanghai, China	0.8679	0.1363	10.658	7.6918	This article

## Data Availability

Not applicable.

## Abbreviations

A glossary of the abbreviated terms used in this article.

Abbreviations	Full Name
AI	artificial intelligence
ANN	artificial neural network
AQGs	Global Air Quality Guidelines
ARIMA	autoregressive integrated moving average
bior	biorthogonal
BP	back propagation
BR	Bayesian regularization algorithm
CA	approximate composition
CD	detailed composition
CNN	convolutional neural network
coif	coiflet
CWT	continuous wavelet transform
DA-RNN	dual-stage attention-based recurrent neural network
db	Daubechies
DL	deep learning
DTR	decision tree regressor
DWT	discrete wavelet transform
EMD-GRU	gated recurrent unit neural network based on empirical mode decomposition
EWV	extreme wind velocity
GA-SVM	combined genetic algorithm and support vector machine
GBDT	gradient boosted decision trees
GBM	gradient boosting machine
GBRT	gradient boosting regression tree
GDP	gross domestic product
GRU	gated recurrent unit neural network
LM	Levenberg–Marquardt algorithm
LSTM	long short-term memory
MAE	mean absolute error
MAP	mean atmospheric pressure
MAT	mean atmospheric temperature
MAXAP	maximum atmospheric pressure
MAXAT	maximum atmospheric temperature
MAXWV	maximum wind velocity
MINAP	minimum atmospheric pressure
MINAT	minimum atmospheric temperature
MINRH	minimum relative humidity
ML	machine learning
MLR	multiple linear regression
MRH	mean relative humidity
MWP	mean water vapor pressure
MWV	mean wind velocity
P	precipitation
PM_2.5_	fine particulate matter
R	correlation coefficient
RE	relative error
ReLU	rectified linear unit
RF	random forest
RMSE	root mean square error
RNN	recurrent neural network
SH	sunshine hours
SVM	support vector machine
SVR	support vector regression
sym	symlet
WANN	wavelet-ANN
W-GRU	wavelet-GRU
WHO	World Health Organization
W-LSTM	wavelet-LSTM
WT	wavelet transformation
XGBoost	extreme gradient boosting

## References

[1] Balogun A.-L., Tella A., Baloo L., Adebisi N. (2021). A review of the inter-correlation of climate change, air pollution and urban sustainability using novel machine learning algorithms and spatial information science. Urban Clim..

[2] Ding A., Nie W., Huang X., Chi X., Sun J., Kerminen V.M., Xu Z., Guo W., Petäjä T., Yang X. (2016). Long-term observation of air pollution-weather/climate interactions at the SORPES station: A review and outlook. Front. Environ. Sci. Eng..

[3] Hu F., Guo Y. (2021). Health impacts of air pollution in China. Front. Environ. Sci. Eng..

[4] Zheng Y., Xue T., Zhao H., Lei Y. (2022). Increasing life expectancy in China by achieving its 2025 air quality target. Environ. Sci. Ecotechnol..

[5] Guo Q., He Z., Wang Z. (2022). Long-term projection of future climate change over the twenty-first century in the Sahara region in Africa under four Shared Socio-Economic Pathways scenarios. Environ. Sci. Pollut. Res..

[6] Yin H., Brauer M., Zhang J., Cai W., Navrud S., Burnett R., Howard C., Deng Z., Kammen D.M., Schellnhuber H.J. (2021). Population ageing and deaths attributable to ambient PM_2.5_ pollution: A global analysis of economic cost. Lancet Planet. Health.

[7] Lelieveld J., Evans J.S., Fnais M., Giannadaki D., Pozzer A. (2015). The contribution of outdoor air pollution sources to premature mortality on a global scale. Nature.

[8] Fowler D., Pyle J.A., Sutton M.A., Williams M.L. (2020). Global Air Quality, past present and future: An introduction. Philos. Trans. R. Soc. A Math. Phys. Eng. Sci..

[9] Murray C.J.L., Aravkin A.Y., Zheng P., Abbafati C., Abbas K.M., Abbasi-Kangevari M., Abd-Allah F., Abdelalim A., Abdollahi M., Abdollahpour I. (2020). Global burden of 87 risk factors in 204 countries and territories, 1990–2019: A systematic analysis for the Global Burden of Disease Study 2019. Lancet.

[10] Wang Y., Shi M., Lv Z., Liu H., He K. (2021). Local and regional contributions to PM_2.5_ in the Beijing 2022 Winter Olympics infrastructure areas during haze episodes. Front. Environ. Sci. Eng..

[11] Geng G., Zheng Y., Zhang Q., Xue T., Zhao H., Tong D., Zheng B., Li M., Liu F., Hong C. (2021). Drivers of PM_2.5_ air pollution deaths in China 2002–2017. Nat. Geosci..

[12] Wang Y., Gao W., Wang S., Song T., Gong Z., Ji D., Wang L., Liu Z., Tang G., Huo Y. (2020). Contrasting trends of PM_2.5_ and surface-ozone concentrations in China from 2013 to 2017. Natl. Sci. Rev..

[13] Zhong J., Zhang X., Gui K., Wang Y., Che H., Shen X., Zhang L., Zhang Y., Sun J., Zhang W. (2021). Robust prediction of hourly PM_2.5_ from meteorological data using LightGBM. Natl. Sci. Rev..

[14] Xue W., Shi X., Yan G., Wang J., Xu Y., Tang Q., Wang Y., Zheng Y., Lei Y. (2021). Impacts of meteorology and emission variations on the heavy air pollution episode in North China around the 2020 Spring Festival. Sci. China Earth Sci..

[15] Zhang H., Jiang Q., Wang J., Li K., Wang F. (2021). Analysis on the impact of two winter precipitation episodes on PM_2.5_ in Beijing. Environ. Sci. Ecotechnol..

[16] Hepburn C., Qi Y., Stern N., Ward B., Xie C., Zenghelis D. (2021). Towards carbon neutrality and China’s 14th Five-Year Plan: Clean energy transition, sustainable urban development, and investment priorities. Environ. Sci. Ecotechnol..

[17] Yang H., Huang X., Hu J., Thompson J.R., Flower R.J. (2022). Achievements, challenges and global implications of China’s carbon neutral pledge. Front. Environ. Sci. Eng..

[18] Barua S., Nath S.D. (2021). The impact of COVID-19 on air pollution: Evidence from global data. J. Clean. Prod..

[19] Wijnands J.S., Nice K.A., Seneviratne S., Thompson J., Stevenson M. (2022). The impact of the COVID-19 pandemic on air pollution: A global assessment using machine learning techniques. Atmos. Pollut. Res..

[20] Habeebullah T.M., Munir S., Zeb J., Morsy E.A. (2022). Modelling the Effect of COVID-19 Lockdown on Air Pollution in Makkah Saudi Arabia with a Supervised Machine Learning Approach. Toxics.

[21] Manoj M.G., Satheesh Kumar M.K., Valsaraj K.T., Vijayan S.K., Nishanth T. (2022). Exacerbation of Fatality Rates Induced by Poor Air Quality Due to Open-Air Mass Funeral Pyre Cremation during the Second Wave of COVID-19. Toxics.

[22] Kaewrat J., Janta R., Sichum S., Rattikansukha C., Tala W., Kanabkaew T. (2022). Human Health Risks and Air Quality Changes Following Restrictions for the Control of the COVID-19 Pandemic in Thailand. Toxics.

[23] Tyagi B., Vissa N.K., Ghude S.D. (2022). Evolution of Pollution Levels from COVID-19 Lockdown to Post-Lockdown over India. Toxics.

[24] Huang X., Ding A., Gao J., Zheng B., Zhou D., Qi X., Tang R., Wang J., Ren C., Nie W. (2020). Enhanced secondary pollution offset reduction of primary emissions during COVID-19 lockdown in China. Natl. Sci. Rev..

[25] Guo Q., Wang Z., He Z., Li X., Meng J., Hou Z., Yang J. (2021). Changes in Air Quality from the COVID to the Post-COVID Era in the Beijing-Tianjin-Tangshan Region in China. Aerosol Air Qual. Res..

[26] Cai W.-J., Wang H.-W., Wu C.-L., Lu K.-F., Peng Z.-R., He H.-D. (2021). Characterizing the interruption-recovery patterns of urban air pollution under the COVID-19 lockdown in China. Build. Environ..

[27] Han J., Yin J., Wu X., Wang D., Li C. (2023). Environment and COVID-19 incidence: A critical review. J. Environ. Sci..

[28] Bao R., Zhang A. (2020). Does lockdown reduce air pollution? Evidence from 44 cities in northern China. Sci. Total Environ..

[29] Jiang X., Wei P., Luo Y., Li Y. (2021). Air Pollutant Concentration Prediction Based on a CEEMDAN-FE-BiLSTM Model. Atmosphere.

[30] Su X., An J., Zhang Y., Zhu P., Zhu B. (2020). Prediction of ozone hourly concentrations by support vector machine and kernel extreme learning machine using wavelet transformation and partial least squares methods. Atmos. Pollut. Res..

[31] Kshirsagar A., Shah M. (2022). Anatomization of air quality prediction using neural networks, regression and hybrid models. J. Clean. Prod..

[32] Masood A., Ahmad K. (2021). A review on emerging artificial intelligence (AI) techniques for air pollution forecasting: Fundamentals, application and performance. J. Clean. Prod..

[33] Saldarriaga J.F., Cruz Y., Estiati I., Tellabide M., Olazar M. (2022). Assessment of pressure drop in conical spouted beds of biomass by artificial neural networks and comparison with empirical correlations. Particuology.

[34] Karimi M., Vaferi B., Hosseini S.H., Olazar M., Rashidi S. (2021). Smart computing approach for design and scale-up of conical spouted beds with open-sided draft tubes. Particuology.

[35] Ma S., Wu T., Chen X., Wang Y., Tang H., Yao Y., Wang Y., Zhu Z., Deng J., Wan J. (2022). An artificial neural network chip based on two-dimensional semiconductor. Sci. Bull..

[36] Wei S., Chen Z., Arumugasamy S.K., Chew I.M.L. (2022). Data augmentation and machine learning techniques for control strategy development in bio-polymerization process. Environ. Sci. Ecotechnol..

[37] Cabaneros S.M., Calautit J.K., Hughes B.R. (2019). A review of artificial neural network models for ambient air pollution prediction. Environ. Model. Softw..

[38] Zhang Y., Yang Q. (2018). An overview of multi-task learning. Natl. Sci. Rev..

[39] Xing J., Zheng S., Ding D., Kelly J.T., Wang S., Li S., Qin T., Ma M., Dong Z., Jang C. (2020). Deep Learning for Prediction of the Air Quality Response to Emission Changes. Environ. Sci. Technol..

[40] Huang G., Ge C., Xiong T., Song S., Yang L., Liu B., Yin W., Wu C. (2021). Large scale air pollution prediction with deep convolutional networks. Sci. China Inf. Sci..

[41] He Z., Zhang Y., Guo Q., Zhao X. (2014). Comparative Study of Artificial Neural Networks and Wavelet Artificial Neural Networks for Groundwater Depth Data Forecasting with Various Curve Fractal Dimensions. Water Resour. Manag..

[42] Guo Q., He Z. (2021). Prediction of the confirmed cases and deaths of global COVID-19 using artificial intelligence. Environ. Sci. Pollut. Res..

[43] Guo Q., He Z., Li S., Li X., Meng J., Hou Z., Liu J., Chen Y. (2020). Air Pollution Forecasting Using Artificial and Wavelet Neural Networks with Meteorological Conditions. Aerosol Air Qual. Res..

[44] He Z., Guo Q., Wang Z., Li X. (2022). Prediction of Monthly PM_2.5_ Concentration in Liaocheng in China Employing Artificial Neural Network. Atmosphere.

[45] Goudarzi G., Hopke P.K., Yazdani M. (2021). Forecasting PM_2.5_ concentration using artificial neural network and its health effects in Ahvaz, Iran. Chemosphere.

[46] Zhou Z.-H., Feng J. (2018). Deep forest. Natl. Sci. Rev..

[47] Li H. (2017). Deep learning for natural language processing: Advantages and challenges. Natl. Sci. Rev..

[48] Iwabuchi K., Kato K., Watari D., Taniguchi I., Catthoor F., Shirazi E., Onoye T. (2022). Flexible electricity price forecasting by switching mother wavelets based on wavelet transform and Long Short-Term Memory. Energy AI.

[49] Wu X., He S., Guo J., Sun W. (2021). A multi-scale periodic study of PM_2.5_ concentration in the Yangtze River Delta of China based on Empirical Mode Decomposition-Wavelet Analysis. J. Clean. Prod..

[50] Mallat S.G. (1989). Multifrequency channel decompositions of images and wavelet models. IEEE Trans. Acoust. Speech Signal Process..

[51] Rene E., Veiga M.C., Kennes C. (2010). Neural network models for biological waste–Gas treatment systems. J. Biotechnol..

[52] MacKay D.J.C. (1992). Bayesian Interpolation. Neural Comput..

[53] Sang Y.F. (2013). A review on the applications of wavelet transform in hydrology time series analysis. Atmos. Res..

[54] Moreno-Barón L., Cartas R., Merkoçi A., Alegret S., del Valle M., Leija L., Hernandez P.R., Muñoz R. (2006). Application of the wavelet transform coupled with artificial neural networks for quantification purposes in a voltammetric electronic tongue. Sens. Actuators B Chem..

[55] Agarwal S., Sharma S., Suresh R., Rahman M.H., Vranckx S., Maiheu B., Blyth L., Janssen S., Gargava P., Shukla V.K. (2020). Air quality forecasting using artificial neural networks with real time dynamic error correction in highly polluted regions. Sci. Total Environ..

[56] Jida S.N., Hetet J.-F., Chesse P., Guadie A. (2021). Roadside vehicle particulate matter concentration estimation using artificial neural network model in Addis Ababa, Ethiopia. J. Environ. Sci..

[57] Wood D.A. (2022). Trend decomposition aids forecasts of air particulate matter (PM_2.5_) assisted by machine and deep learning without recourse to exogenous data. Atmos. Pollut. Res..

[58] Gui K., Che H., Zeng Z., Wang Y., Zhai S., Wang Z., Luo M., Zhang L., Liao T., Zhao H. (2020). Construction of a virtual PM_2.5_ observation network in China based on high-density surface meteorological observations using the Extreme Gradient Boosting model. Environ. Int..

[59] Kim D., Ho C.-H., Park I., Kim J., Chang L.-S., Choi M.-H. (2022). Untangling the contribution of input parameters to an artificial intelligence PM_2.5_ forecast model using the layer-wise relevance propagation method. Atmos. Environ..

[60] Guo X., Wang Y., Mei S., Shi C., Liu Y., Pan L., Li K., Zhang B., Wang J., Zhong Z. (2022). Monitoring and modelling of PM_2.5_ concentration at subway station construction based on IoT and LSTM algorithm optimization. J. Clean. Prod..

[61] Jiang Z., Zheng T., Bergin M., Carlson D. (2022). Improving spatial variation of ground-level PM_2.5_ prediction with contrastive learning from satellite imagery. Sci. Remote Sens..

[62] Huang G., Li X., Zhang B., Ren J. (2021). PM_2.5_ concentration forecasting at surface monitoring sites using GRU neural network based on empirical mode decomposition. Sci. Total Environ..

[63] Huang C.-J., Kuo P.-H. (2018). A Deep CNN-LSTM Model for Particulate Matter (PM_2.5_) Forecasting in Smart Cities. Sensors.

[64] Faraji M., Nadi S., Ghaffarpasand O., Homayoni S., Downey K. (2022). An integrated 3D CNN-GRU deep learning method for short-term prediction of PM_2.5_ concentration in urban environment. Sci. Total Environ..

[65] Liu H., Long Z., Duan Z., Shi H. (2020). A New Model Using Multiple Feature Clustering and Neural Networks for Forecasting Hourly PM_2.5_ Concentrations, and Its Applications in China. Engineering.

[66] Luo Z., Huang F., Liu H. (2020). PM_2.5_ concentration estimation using convolutional neural network and gradient boosting machine. J. Environ. Sci..

[67] Du W., Chen L., Wang H., Shan Z., Zhou Z., Li W., Wang Y. (2023). Deciphering urban traffic impacts on air quality by deep learning and emission inventory. J. Environ. Sci..

[68] Zhang P., Ma W., Wen F., Liu L., Yang L., Song J., Wang N., Liu Q. (2021). Estimating PM_2.5_ concentration using the machine learning GA-SVM method to improve the land use regression model in Shaanxi, China. Ecotoxicol. Environ. Saf..

